# PGE_2_ Promotes Apoptosis Induced by Cytokine Deprivation through EP3 Receptor and Induces Bim in Mouse Mast Cells

**DOI:** 10.1371/journal.pone.0102948

**Published:** 2014-07-23

**Authors:** Martina Kovarova, Beverly H. Koller

**Affiliations:** 1 Department of Medicine, Pulmonary Division, University of North Carolina, Chapel Hill, Chapel Hill, North Carolina, United States of America; 2 Department of Genetics, University of North Carolina, Chapel Hill, Chapel Hill, North Carolina, United States of America; Virginia Tech University, United States of America

## Abstract

Increased mast cell numbers are observed at sites of allergic inflammation and restoration of normal mast cell numbers is critical to the resolution of these responses. Early studies showed that cytokines protect mast cells from apoptosis, suggesting a simple model in which diminished cytokine levels during resolution leads to cell death. The report that prostaglandins can contribute both to recruitment and to the resolution of inflammation together with the demonstration that mast cells express all four PGE_2_ receptors raises the question of whether a single PGE_2_ receptor mediates the ability of PGE_2_ to regulate mast cell survival and apoptosis. We report here that PGE_2_ through the EP3 receptor promotes cell death of mast cells initiated by cytokine withdrawal. Furthermore, the ability of PGE_2_ to limit reconstitution of tissues with cultured mast cells is lost in cell lacking the EP3 receptor. Apoptosis is accompanied by higher dissipation of mitochondrial potential (ΔΨ_m_), increased caspase-3 activation, chromatin condensation, and low molecular weight DNA cleavage. PGE_2_ augmented cell death is dependent on an increase in intracellular calcium release, calmodulin dependent kinase II and MAPK activation. Synergy between the EP3 pathway and the intrinsic mitochondrial apoptotic pathway results in increased Bim expression and higher sensitivity of mast cells to cytokine deprivation. This supports a model in which PGE_2_ can contribute to the resolution of inflammation in part by augmenting the removal of inflammatory cells in this case, mast cells.

## Introduction

Mast cells are long-lived tissue resident cells found throughout the body primarily in association with blood vessels, nerves, and in proximity to surfaces that interface the external environment. Upon activation, mast cells release inflammatory mediators, including histamine, proteases, prostaglandins, leukotrienes and cytokines [1]. Mast cells have an important function in several physiological as well as pathophysiological processes including host defense, especially in response to parasites, allergic reaction and inflammation. It is, therefore not surprising, that mast cell numbers increase at sites of inflammation during the course of the response. For example, elevated numbers of mast cells are observed in the submucosa of the lungs of asthmatics [2],[3], allergy [4], rheumatoid arthritis [5], [6], and chronic allergic dermatitis [6].

During the resolution phase of inflammation, homeostasis is reestablished in inflamed tissues and mast cell numbers decline. For many immune cells, survival at the site of inflammation is enhanced by cytokines, and therefore a decrease in local levels of these mediators, as the threat to the organism is neutralized, can lead to apoptosis. “Cytokine withdrawal” has been reported to activate an intrinsic (mitochondrial) apoptotic pathway in immune cells resulting in compromised mitochondrial integrity [7]. The mitochondrial integrity is guarded by Bcl-2 protein family members including anti-apoptotic proteins Bcl-2, Bcl-X_L_, Mcl-1, A1 and pro-apoptotic proteins Bax, Bak, Bim, Bid, Puma, Noxa, Bad, Bik, Bmf and Hrk. Mitochondrial outer membrane permeabilisation (MOMP) occurs when the balance of these factors is markedly disturbed. MOMP results in the release of principal killing factors such as cytochrome c and Smac/DIABLO from mitochondria to cytoplasm, where they contribute to the formation of apoptosome and activate aspartate-specific cysteine proteases (caspases) including initiator caspase-9. Caspase-9 in turn cleaves and induces the activation of downstream effector caspases that degrade and disassemble the cell [7].

Mast cell survival is regulated primarily by Stem cell factor (SCF), the ligand of c-kit receptor, through inactivation of the Forkhead transcription factor FOXO3a by MEK/MAPK- and PI3-kinase-mediated phosphorylation. Phosphorylation of FOXO3a also leads to phosphorylation, subsequent ubiquitination and proteasomal degradation of proapoptotic Bim and Puma. Upon cytokine withdrawal, phosphorylation of FOXO3a decreases, followed by increase in Bim and Puma expression and apoptosis [8].

Prostaglandin E_2_ (PGE_2_), a bioactive mediator elevated at sides of inflammation, exerts its biological function through four distinct membrane-bound G-coupled receptors EP1- EP4. PGE_2_ can contribute to resolution of inflammation by stimulating the expression of lipid mediators involved in the regulation of phagocytic clearance of apoptotic cells and by suppressing of the initial inflammatory response [9]. Here we examine the ability of PGE_2_ to contribute to the resolution of inflammation, specifically, the removal of mast cells from inflammatory sites.

## Material and Methods

### Chemicals

LY294002, PD98059, PGE_2_, KN-93 were from Cayman (Ann Arbor, MI, USA). All other chemicals were from Sigma-Aldrich (St. Louis, MO, USA).

### Mice

The generation of mice deficient in the EP_1_, EP_2_, EP_3_, and EP_4_ receptors and mPGES1 has been previously reported [10]–[14]. All mice used were at least 8 wks old and were bred and maintained in specific pathogen-free animal facilities at the University of North Carolina (Chapel Hill, NC). Mice were killed by exposure to CO_2_ follow by physical euthanasia prior to collection of cells. All experiments were carried out in accordance with the recommendations in the Guide for the Care and Use of Laboratory Animals of the National Institutes of Health. The protocol was approved by the Institutional Animal Care and Use Committee guidelines of the University of North Carolina (permit number:13–158).

### Mast cell culture and cytokine depletion

Mast cells were derived from bone marrow isolated from mouse tibia and cultured in complete medium (Iscove's Modified Dulbecco's Medium supplemented with 12% fetal bovine serum (FBS), 100 U/ml of Penicillin and Streptomycin, 4 mM L-glutamine, 10 mM HEPES, 1 mM sodium pyruvate, 100 µM non-essential amino acid (all from GIBCO Life Technologies, Grand Island, NY, USA)), 10 ng/ml of recombinant mouse IL-3 and 20 ng/ml of recombinant mouse SCF (both from Sigma-Aldrich, St. Louis, MO, USA) for 4 weeks at 37°C in a humidified incubator with 5% CO_2_ atmosphere. Medium was changed weekly and adherent cells were removed regularly from cultures by transfer of non-adherent cells to a new culture dish. Maturity of non-adherent BMMC was assessed by measuring surface expression of FcεRI and c-Kit. Only cultures with >95% cells positive for both receptors and <12 weeks in culture were used for experiments.

For depletion, BMMC were washed twice in phosphate buffered saline (PBS) and re-suspended in depletion medium (the same as the culture medium but without IL-3 and SCF) at a concentration 1×10^6^ cells per 1 ml. Cells were cultured at 37°C in a humidified incubator with 5% CO_2_ atmosphere.

### Cell death and apoptosis analysis

#### 7AAD

Cells depleted of cytokines for various times were stained for 15 min in 7-AAD (BD Bioscience, Franklin Lakes, NJ USA), 5 µl per test (1×10^6^ cells) and analyzed using Fluorescence-activated cell sorting (FACS) on a CyAn ADP analyzer (Beckman Coulter, Inc., Brea, CA).

#### Mitochondrial membrane potential

Cells were stained using JC-1 from a Flow cytometry mitochondrial membrane potential detection kit (BD Biosciences, Franklin Lakes, NJ USA) according to the manufacturer's protocol. Green and red fluorescence ratios were measured on CyAn ADP analyzer (Beckman Coulter, Inc., Brea, CA).

#### Caspase-3 activation

Caspase activation was measured with the APO LOGIX carboxyfluorescein (FAM) caspase detection kit (Cell Technology, Inc., Mountain View, CA, USA) specific for caspase-3 (FAM500-2) according to the manufacturer's protocol. Fluorescence was measured on a CyAn ADP analyzer (Beckman Coulter, Inc., Brea, CA) and results were verified by western blot using an antibody specific for cleaved caspase-3 antibody (#9661, Cell Signaling Technology, Danvers, MA, USA).

#### Low molecular weight cleavage assessment

3×10^6^ BMMC depleted of cytokines for 24 h were lysed in lysis buffer (1% SDS, 0.1 M NaCl, 0.1 M EDTA, 0.05 M Tris base, 0.5 mg/ml proteinase K, 0.1 mg/ml RNase A, pH 8) for 2 h at 55°C. 135 µl of saturated NaCl solution was added to each sample, mixed and centrifuged for 10 min at 16,000 g. Supernatant (400 µl) was removed, added to 800 µl 100% ethanol and precipitated for 20 min at −20°C. Precipitated DNA was pelleted by centrifugation at 16,000 g and washed with 0.5 ml 70% ethanol and 0.5 ml 100% ethanol. The pellet of DNA was air dried for 10 min at room temperature to remove trace of ethanol and then dissolve in 30 µl TE buffer (10 mM Tris base, 1 mM EDTA). Loading buffer was then added. Samples were separated by electrophoreses on 1.2% agarose gel 5 V/cm for 1–2 h. The gel was stained with ethidium bromide and visualized by trans-illumination with UV light and photographed.

#### Condensed nuclei staining

BMMC depleted of cytokines for 24 h were placed on poly-L-lysine-treated coverslips, fixed with 4% paraformaldehyde (Sigma-Aldrich, St. Louis, MO, USA) for 10 min at room temperature, and mounted on microscope slides (Thermo Fisher Scientific Inc., Waltham, MA, USA) in 5 µl of ProLong Gold antifade reagent with 4,6-diamidino-2-phenylindole (Invitrogen, Life Technologies, Grand Island, NY, USA). Samples were visualized using an Olympus BX61 upright fluorescence microscope (Olympus, Center Valley, PA, USA) with a 60× objective with a Hamamatsu ORCA RC camera (Hamamatsu Photonics, K.K., Hamamatsu City, Japan), operated by Velocity software (PerkinElmer Life and Analytical Sciences, Waltham, MA, USA).

### Isolation of peritoneal mast cells

Peritoneal cells from 5 congenic mice were pooled and washed once with PBS. Cells were re-suspended in 8 ml of 70% Percoll solution (7 ml Percoll (GE healthcare Bioscience AB, Upsala, Sweden), 1 ml 10× concentrated PBS, 1.9 ml H_2_O, 0.1 ml FBS) and overlaid with 2 ml of peritoneal mast cell medium (PMC: DMEM, 20 mM HEPES, 5% FBS). Cells were centrifuged 650 g at room temperature for 15 min. Supernatant was discarded and mast cell pellets were re-suspended in 0.5 ml PMC medium, transferred to new tubes and washed once in 10 ml pre-warmed PMC medium.

### Intracellular calcium measurement

BMMC in complete culture medium were harvested, washed and resuspended in Hank's balanced salt solution buffer with Ca++, Mg++ and 0.1% BSA (HBSS, GIBCO Invitrogen) at a concentration of 1×10^6^cells/ml. Cells were loaded with 2 µM FURA-2/AM (Invitrogen) for 40 min, and washed twice. Changes in dye fluorescence (excitation 340 and 380 nm, emission 510 nm) with time after BMMC stimulation with 1×10^−6^ M PGE_2_ were determined by Fluostar Optima spectrometer (BMG Labtechnologies). Calcium concentrations were calculated as described previously [15]. For inhibition of intracellular calcium with BAPTA was added to cell suspension 5 min before stimulation with PGE_2_.

### Immunoblotting analysis

Whole cell lysates in SDS-PAGE loading buffer were fractioned by SDS-PAGE under reducing condition and electrotransferred to PVDF membrane (Hybond-P, Amersham, GE Healthcare, Uppsala, Sweden). The following primary antibodies were used for immunostaining of the membranes: caspase-3 (#9661), phospho-Erk1/2 (#9106), phospho-Akt (#5171), phospho-p38 (#9211), c-Jun (#9165), Bim (#2933) (all from Cell Signaling Technology (Danvers, MA, USA)), β-actin (A5316, Sigma-Aldrich, St. Louis, MO, USA). The secondary antibodies anti mouse-HRP (#7076) anti rabbit-HRP (#7074) from Cell Signaling Technology (Danvers, MA, USA) were utilized. All immunostaining was done according to the manufacturer's protocol. Results were analyzed by a gel analysis module in the ImageJ software (NIH, http://imagej.nih.gov/ij/index.html).

### Gene expression assay

RNA was isolated with RNAbee (Tel-Test Inc., Friendswood, TX) according to the manufacturer's protocol and cleaned with the Total RNA mini purification kit (Denville Scientific Inc., Metuchen, NJ, USA). Reverse transcription was carried out with the High Capacity cDNA transcription kit (Applied Biosystems, Life Technologies, Grand Island, NY, USA). Obtained cDNAs were used for relative quantitative analysis by real-time PCR using comparative CT method. The following primers and detection TaqMan MGB probes were used (*Bcl2l11* (Bcl-2-like protein 11/Bim) # Mm00437796, *Bbc3* (Bcl-2-binding component 3/Puma) #Mm00519268) for cDNA amplification by ABI Prism 7 900HT detection system in TaqMan universal PCR master mix (all from Applied Biosystems, Life Technologies, Grand Island, NY, USA) according to the manufacturer's protocol. The gene expression level was expressed as a relative expression of the gene of interest to GAPDH expression level in each sample.

### Reconstitution of mast cells in ear of W^sh/sh^ mice

W^sh/sh^ mice were lightly anesthetized and the pinna of the ears reconstituted intradermally (i.d.) with various numbers of 5-week-old cultured BMMC in 40 µl PBS. In other experiments mice received intradermally to both ears 5×10^5^ BMMC, treated or not with 1×10^−6^ M PGE_2_ for 20 min and washed twice with PBS to remove PGE_2_ before injection.

### Passive cutaneous anaphylaxis

Animals were lightly anesthetized, and the pinna of the right ears injected i.d. with 8 ng of murine monoclonal anti-DNP IgE in 20 µl of PBS. The left ears received 20 µl of PBS, i.d. Twenty-four hours later, animals were injected i.v. with 100 µl of PBS containing 100 µg of DNP-albumin and 1% Evan's Blue dye. Animals were killed 90 min after *i.v.* injection, and pinna of the ears were removed close to the base and incubated in 1 ml of formamide at 54°C for 48 h. Quantitative analysis of formamide extracts was determined by measuring the absorbance of Evan's Blue at 610 nm with a spectrophotometer.

### Statistical analysis

Data are represented as means ± SEM. Statistical significance was assessed by the Student's two-tailed t test. When three or more groups or variables were compered, statistical significance was determined by ANOVA. A P value of <0.05 was considered as statistically significant

## Results

### PGE_2_ increases cell death during cytokine deprivation

**Figure 1:**
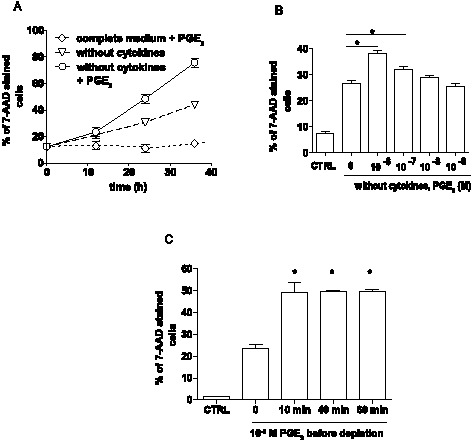
PGE_2_ promotes mast cell death during cytokine deprivation. **A**. The percentage of 7-AAD stained mast cells after culture in complete medium with 1×10^−6^ M PGE_2_, in depletion medium without cytokines and in depletion medium with 1×10^−6^M PGE_2_. Data are from 8 independent experiments using 2 cultures of WT BMMC. **B**. The percentage of 7AAD stained cells during cytokine deprivation for 16 h carried out in the presence of various PGE_2_ concentrations. Data are from 8 independent experiments using 1 culture of WT BMMC. **C**. 7-AAD positive BMMC pretreated with 1×10^−6^ M PGE_2_ in complete medium for various times. Cells were depleted from cytokines for 16 h without further presence of PGE_2_. Data are from 4 independent experiments using 1 culture of WT BMMC. CTRL indicates mast cells cultured in complete medium during the experiment. ANOVA was used in B and C to evaluate statistical significance. Statistical significance: * = P<0.05, ** = P<0.01.

Interleukin-3 (IL-3) and SCF are critical to mast cell survival, both *in vitro* and *in vivo*. We took advantage of this to model cell death of mast cells *in vitro*. Mast cells derived from bone marrow of C57BL/6J mice (BMMC) in complete medium containing IL-3 and SCF, were deprived of these cytokines to initiate cell death, either in the presence or absence of PGE_2_. Cell death was evaluated as a percentage of 7-AAD-stained cells at various times after cytokine withdrawal. Cytokine deprivation resulted in an increase in the number of 7-AAD-stained cells, reaching 30% 24 h and 44% 36 h after cytokine removal. Addition of 1×10^−6^ M PGE_2_ to the cytokine depleted medium led to a further increase in number of 7-AAD stained cells compared to untreated BMMC with 48% and 76% of the cells staining with the dye 24 h and 36 h after cytokine removal, respectively. Nevertheless, addition of 1×10^−6^ M PGE_2_ to complete medium did not result in an increase of 7-AAD stained cells (Fig.1A). The difference in numbers of 7-AAD-stained cells during cytokine withdrawal in the presence or absence of PGE_2_ was significant (P<0.05) in concentrations from 1×10^−6^ M to 1×10^−7^ M (Fig 1B). Interestingly, pretreatment of mast cells with PGE_2_ for only 10 min in complete medium follow by cytokine deprivation without further addition of PGE_2_ to the medium was sufficient to increase the number of 7-AAD positive cells to a similar magnitude as observed when PGE_2_ was present throughout the experiment (Fig 1C). This suggests that PGE_2_ can affect cell survival even after its concentration return to homeostatic levels.

### Increased cell death after PGE_2_ treatment is caused by apoptosis

**Figure 2:**
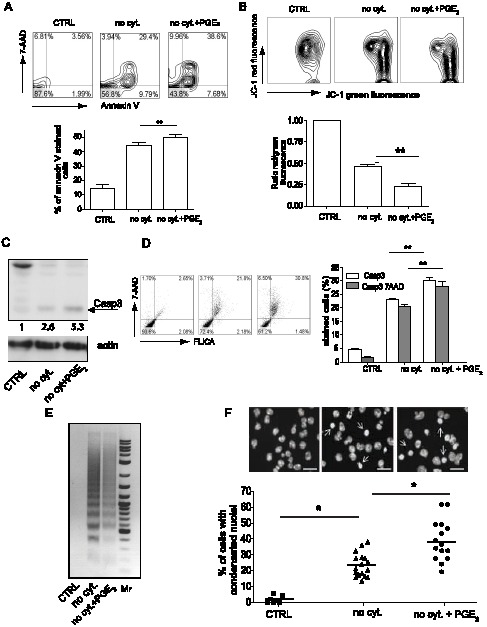
PGE_2_ increases apoptosis induced by cytokines withdrawal. **A**. Double staining of BMMC with 7-AAD and APC-Annexin V (upper panel), and its quantitative analysis (lower panel). Data are from 8 independent experiments using 2 cultures of WT BMMC. **B**. FACS analysis of the mitochondrial potential measured as fluorescence intensity of JC-1 staining (upper panel), and its quantitative analysis expressed as a ratio of red fluorescence (FL-2 channel) and green fluorescence (FL-1 channel). Ratios were normalized to CTRL samples in each experiment (lower panel). Data are from 5 independent experiments using 1 culture of WT BMMC. **C**. Representative western blot analysis from 3 independent experiments using 1 culture of WT BMMC of caspase-3 activation measured by the presence of a cleaved p17 fragment in BMMC (upper panel), arrow indicates p17 fragment. Anti-actin antibody was used as a loading control (lower panel). **D**. FACS analysis of BMMC stained with 7-AAD and FLICA for caspase-3 activity (left panel), and its quantitative analysis (right panel). Data are from 5 independent experiments and 1 culture of WT BMMC. **E**. Agarose gel analysis of the DNA fragmentation assay in BMMC. Mr stands for 2-Log DNA Ladder. Data are from 3 independent experiment and 1 culture of WT BMMC. **F**. Chromatin condensation in BMMC stained with DAPI (upper panel), and quantitative analysis (lower panel). Figures were captured by an Olympus BX61 upright fluorescence microscope with 40× objectives with a Hamamatsu ORCA RC camera, operated by Velocity software (PerkinElmer Life and Analytical Sciences). Bars in insets represent 10 µm; arrows show condensed nuclei. Data are from 15 independent experiments using 3 culture of WT BMMC. Samples: BMMC cultured in complete medium (CTRL), BMMC cultures in medium without cytokines (no cyt.), BMMC treated with 10^−6^ M PGE_2_ and cultured in medium without cytokines (no cyt. + PGE_2_). Student's two-tailed t test was used to evaluate statistical differences between cytokine deprive mast cells and cytokine deprived mast cells treated with PGE_2_ in B and D. Statistical significance: * = P<0.05, ** = P<0.01.

To further characterize the PGE_2_ mediated increase in BMMC death, we measured loss of plasma membrane integrity and extracellular translocation of phosphatidylserine (PS) with APC-labeled Annexin V. Twenty hours after cytokine withdrawal, about 40% of BMMC stained with Annexin V. Treatment with PGE_2_ increased this number to 45% of BMMC (Fig. 2A). A characteristic feature of cell apoptosis is a loss of mitochondrial membrane potential (ΔΨ_m_), which can be measured by cationic dye JC-1 as a potential-dependent ratio of red and green fluorescence intensity. As shown in Fig. 2B, the decrease of red fluorescence intensity, a sign of dissipation of ΔΨ_m_ during cytokine deprivation, was significantly higher in PGE_2_ treated BMMC compared to un-treated BMMC. Caspase-3 is a critical executioner of apoptosis. Its activation requires proteolytic processing of the inactive zymogen into activated p17 and p12 fragments. Western blot analysis with antibody specific to the cleaved p17 fragment showed higher levels of this fragment in lysates prepared from the cytokine deprived PGE_2_ treated BMMC compared to similar cultures without PGE_2_ (Fig 2C). Consistent with this, PGE_2_ treatment of cytokine-deprived cells increased the number of BMMC that stained with the fluorescently labeled inhibitor of caspase-3 (FLICA) FAM-DEVD-FMK (Fig 2D). Similarly to double staining with Annexin V in Fig. 2A, the majority of 7-AAD-stained cells are also stained with FLICA (Fig. 2D), suggesting that the increase in cell death shown in Fig 1 is directly related to increase in caspase-3 activation and apoptosis.

Characteristic hallmarks of the late phase of apoptotic execution are low molecular weight cleavage (LMW) of DNA and chromatin condensation. Cytokine deprivation of both PGE_2_ treated and untreated BMMC showed a pattern of DNA fragmentation consistent with apoptosis (Fig 2E). Similarly, the number of cells with condensed chromatin was higher in BMMC treated with PGE_2_ compare to untreated BMMCs (Fig 2F). The observation of multiple events characteristic of apoptosis in BMMC after cytokine withdrawal, and the increase in the magnitude of these changes in PGE_2_ treated cells, is consistent with a model in which PGE_2_ augments the apoptotic pathway initiated by cytokine withdrawal.

### PGE_2_ activation of the EP3 receptor increases apoptosis

**Figure 3:**
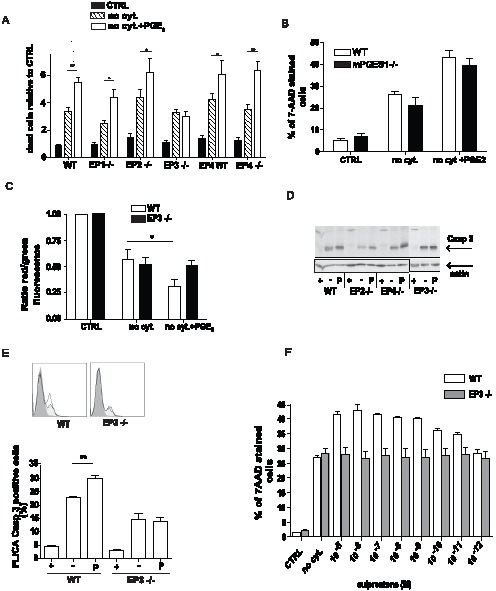
EP3 is responsible for PGE_2_-increased apoptosis. **A**. PGE_2_-mediated increase in mast cell death evaluated by 7-AAD staining was determined for BMMCs lacking each of the four PGE_2_ receptor. Data are from 5 independent experiments using 2 cultures of each genotype. **B**. PGE_2_ mediated increase in apoptosis in mPGES1−/− BMMC. Dead cells numbers were determined by 7-AAD staining. Data are from 3 independent experiments and 1 culture of BMMC per genotype. C. Decreased mitochondrial membrane potential in WT and EP3 −/− BMMC measured as a relative ratio of red and green fluorescence of JC-1 dye. Data are from 5 independent experiments using 1 culture of WT and EP3 −/− BMMC. **D**. Activity of caspase-3 in WT, EP2, EP3, and EP4 deficient BMMC identified by specific antibody to cleaved p17 caspase-3 fragment. Data are representative of 3 experiments for EP2−/− and EP4−/− and 4 experiments for WT and EP3−/− using 1 culture of each genotype. **E**. Staining by caspase 3 specific FLICA; example of FACS histogram (upper panel) and its quantitative analysis (lower panel). Data are from 4 independent experiments of 1 culture of WT BMMC and EP3−/−. BMMC were cultured in complete medium (CTRL or +), medium without cytokines (no cyt. or -) and medium without cytokines in the presence 1×10^−6^ M PGE_2_ (no cyt. + PGE_2_, or P) for 16 h. Student's two-tailed t test was used to evaluate statistical differences between cytokine deprive mast cells and cytokine deprived mast cells treated with PGE_2_ in A, C, and E. Statistical significance: * = P<0.05, ** = P<0.01. F. Increase in mast cell death after 20 min treatment with various concentration of sulprostone. Cells cultured for 16 h in complete medium (CTRL), medium without cytokines (no cyt.) and stain with 7AAD. Data from 3 independent experiments of 1 culture of WT BMMC.

BMMC express all four PGE_2_ receptors [16]. Therefore BMMC were derived from mice lacking each of these receptors and the appropriate controls. The impact of the loss of each of the four PGE_2_ receptors on the ability of 1×10^−6^ M PGE_2_ to augment apoptosis initiated by cytokine withdrawal was assessed by determining the number of 7AAD positive cells in the cultures 20 hours after initiation of the experiment. The absence of the EP1, EP2 or EP4 receptors did not alter the augmentation of 7AAD positive cells in the PGE_2_ treated cultures (Fig. 3A). In contrast, no increase in the number of 7AAD staining cells was observed in PGE_2_ treated EP3−/− BMMC deprived of cytokines, compared to untreated cultures (Fig 3A). During the experiment we noted some variability between different clones of mast cells. Although, mast cells generally do not produce large amounts of PGE_2_, it is possible that during cytokine deprivation and cell death, endogenous PGE_2_ is released and thus PGE_2_ produced by the mast cell itself augments apoptosis. This can contribute to differences in cell death seen in various mast cell cultures. To determine the contribution of such an autocrine loop to mast cell apoptosis, BMMC were derived from mice lacking mPGES1 (mPGES1−/−), the primary PGE_2_ synthase. mPGES1−/− BMMC showed a similar rate of cell death during cytokine withdrawal and were equally sensitive to a PGE_2_ mediated increase in cell death compare to wild type (WT) cells (Fig 3B). These results show that in this model exogenous rather than endogenous PGE_2_ mediates the increase in mast cell death during cytokine withdrawal.

The absence of EP3 on BMMC eliminated PGE_2_ mediated augmentation of the loss in mitochondrial membrane potential during cytokine depletion (Fig 3C). The activity of caspase-3, measured by presence of the cleaved p17 fragment after cytokine depletion, was not increase by PGE_2_ treatment of EP3−/− BMMC, while WT, EP2−/−, and EP4−/−cells showed higher levels of caspase-3 activation (Fig. 3D). Similarly, caspase-3 activity measured by FLICA staining showed no increase in EP3−/−BMMC when treated with PGE_2_ (Fig.3E). Sulprostone, a metabolism resistant synthetic analog of PGE_2_, is a EP3 receptor preferring agonist. Pretreatment of mast cells with various concentration of sulprostone in complete medium prior to cytokine depletion resulted in concentration dependent increase in cell death measure by 7-AAD. The similar actions of PGE_2_ and sulprostone support the role of EP3 in mast cell apoptosis following cytokine withdrawal (Fig. 3F).

### *Ex vivo* and *in vivo* evaluation of mast cell survival after exposure to PGE_2_

**Figure 4:**
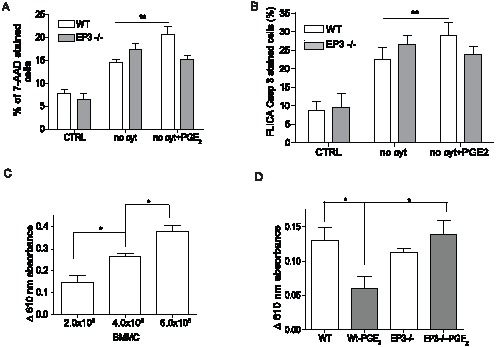
EP3 dependent apoptosis increase in peritoneal mast cells and *in vivo*. **A**. Peritoneal mast cells (PMC) were isolated from the peritoneum of WT and EP3 −/− mice and culture for 16 h in medium with (CTRL) or without (no cyt.) cytokines or with 1×10^−6^ M PGE_2_ (no cyt + PGE_2_). Cells were stained with 7-AAD. Results are from 8 independent experiments. **B**. Staining of PMC from WT and EP3−/− mice with FLICA specific for caspase 3. Results are from 4 independent experiments. **C**. Indicated numbers of BMMC were injected to pinna of the ear of WT mice. 10 day after injection, passive cutaneous anaphylaxis was quantified by assessing serum protein extravasation into tissue in Evans blue treated animals. n = 3 animals for each group. **D**. 5×10^5^ BMMC of the indicated genotypes were treated with 1×10^−6^ PGE_2_ or vehicle for 20 min, washed 2× with PBS and injected to pinna of the ears of mast cells deficient mice (W^sh/sh^). 10 day after injection, passive cutaneous anaphylaxis was assessed as in C. n = 4 animals for each group. Student's two-tailed t test was used to evaluate statistical differences between cytokine deprive mast cells and cytokine deprived mast cells treated with PGE_2_ in A and B. Statistical significance of differences in C and D were evaluated using ANOVA. Statistical significance: * = P<0.05, ** = P<0.01.

BMMCs are widely used as a model for studying mast cells *in vitro*; however they are considered less mature compared to residential mast cells. To verify our finding in primary mast cells, peritoneal mast cells (PMC) from WT and EP3 deficient animals were isolated on Percoll gradient with more than 98% efficiency as describe previously [17]. PMC were cultured either in complete medium, in cytokine-depleted medium, or in cytokine-depleted medium with 1×10^−6^ M PGE_2_ for 16 h. Viability was assessed by determining the number 7AAD positive cells. As expected, a marked increase in the number of 7-AAD staining cells was observed in cytokine-deprived cultures. Similarly to BMMCs the number of 7-AAD cells in the cytokine deprived cultures was significantly increased by addition of PGE_2_. This action of PGE_2_ was dependent on the EP3 receptor as PGE_2_ treatment did not increase the number of 7-AAD stained cells in PMC isolated from EP3 deficient animals (Fig 4A). PGE_2_ treatment of WT PMC, but not EP3−/− PMC also increased the number of cells staining with caspase-3 specific FLICA compared to untreated cells (Fig. 4B).

Study of the role of PGE_2_ in apoptosis *in vivo* is complicated by the fact that in healthy tissue PGE_2_ may act to recruit circulating mast cells progenitors and this action may also be mediated by the EP3 receptor [18], [19]. After PGE_2_ treatment of mice it is not possible to distinguished cells mobilized to the site towards the PGE_2_ gradient and cells already present at the site of PGE_2_ application. Use of sulprostone does not alleviate the problem, since some of the pro-migratory actions of PGE_2_ are mediated through the EP3 receptor. Therefore, to begin to address the *in vivo* role of EP3 in apoptosis, we developed the following model. The skin of mast cells deficient mice (W^sh/sh^) is reconstituted locally by injection of excessive numbers of BMMC, with the anticipation that only small number of cells will survive and stably repopulate tissue. We use the decrease in the amount of transferred BMMC to the ear of W^sh/sh^ mice to evaluate the pro-apoptotic actions of PGE_2_ and assess the ability of the EP3 pathway to contribute to mast cell death *in vivo*. EP3−/− and wild type BMMC were briefly exposed to PGE_2_ washed twice with PBS and then used to reconstitute the pinna of the ear of mast cells deficient W^sh/sh^ mice. After 10 days the survival of the mast cells was compared indirectly by examining the susceptibility of the tissue to suboptimal passive cutaneous anaphylaxis. PCA mediated by exogenous IgE is entirely dependent on mast cells [20]. Accordingly, the extravasation of serum proteins characteristic of this response shows a linear relationship with the number of mast cells introduced into tissue (Fig. 4C). Treatment of wild type but not EP3−/− BMMC with PGE_2_ decreased the anaphylactic response in the reconstituted animals (Fig. 4D). This decrease is consistent with a decrease in survival of mast cells after activation of the EP3 receptor in tissue lacking inflammatory cytokines.

### PGE_2_ mediated increase in apoptosis is dependent on intracellular calcium release and activation of MAPK

To identify the pathway(s) responsible for the PGE_2_ dependent increase in apoptosis during cytokine withdrawal, we compared activation of EP3−/− and WT mast cells, identified pathways activated through this receptor and then evaluated the potential of each of these pathways to contribute to mast cell apoptosis. As we have shown, PGE_2_ treatment prior to cytokine removal can augment apoptosis (Fig.1C). We used this protocol to avoid complexities in studying PGE_2_ pathways after cytokine withdrawal.

**Figure 5:**
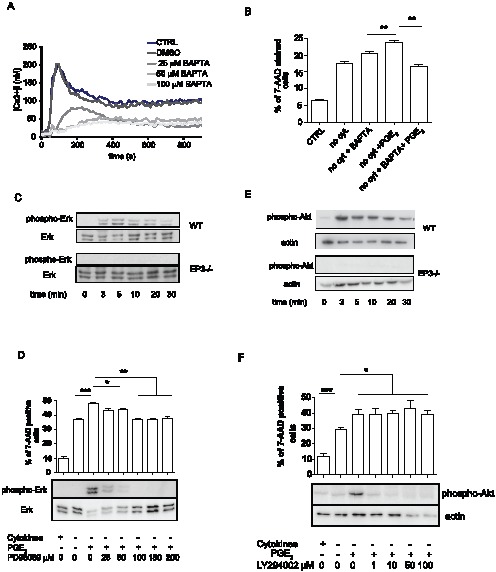
PGE_2_-driven increase in apoptosis is dependent on intracellular calcium release and activation of MAPK. **A**. Intracellular calcium release after stimulation with 1×10^−6^ M PGE_2_ in untreated BMMC or in BMMC treated with various concentrations of BAPTA. Data is representative of 3 independent experiments of 1 WT BMMC culture. **B**. Ability of BAPTA to inhibit PGE_2_ mediated augmentation of cell apoptosis after cytokine withdrawal. Cell were treated as indicated with 25 µM BAPTA for 5 min follow by 1×10^−6^ M treatment with PGE_2_ for 20 min. Cell death was assessed at 16 h of depletion by staining with 7AAD. Data are from 5 independent experiments using 1 culture of WT BMMC. **C**. Time course of Erk1/2 phosphorylation in WT and EP3−/− BMMC stimulated with 1×10^−6^ M PGE_2_, representative of 3 independent experiments is shown. **D**. Pharmacological inhibition of PGE_2_-triggered Erk1/2 phosphorylation (lower panel) and increase in apoptosis (upper panel). BMMC were activated by 1×10^−6^ M PGE_2_. For inhibition, cells were pretreated with various concentrations of PD98059 for 10 min before PGE_2_ activation, n = 5 independent experiment of 2 culture of BMMC. **E**. Time course of Akt phosphorylation in WT and EP3−/− BMMC stimulated with 1×10^−6^ M PGE_2_, n = 3 independent experiment of 1 culture. **F**. Pharmacological inhibition of Akt phosphorylation triggered by PGE_2_ (lower panel) and increase in apoptosis (upper panel), BMMC were activated by 1×10^−6^ M PGE_2_. For inhibition, cells were pretreated with various concentration of LY294002 for 10 min before PGE_2_ activation, n = 6 independent experiments of 2 culture of BMMC. Differences between groups in B were evaluated by Student's two-way t-test, for D and F ANOVA test was used. Statistical significance: * = P<0.05, ** = P<0.01, *** = P<0.001.

Increase in intracellular calcium mobilization after mast cell activation by PGE_2_ is exclusively dependent on the expression of the EP3 receptor [16]. To assess the role of intracellular calcium in apoptosis, we treated BMMC with 100 µM, 50 µM, or 25 µM of BAPTA/AM, a cell-permeating calcium chelator, for 5 min prior to EP3 receptor activation with 1×10^−6^ M PGE_2_. Treatment of cells with either 50 µM or 100 µM BAPTA completely inhibited intracellular calcium release and partial inhibition of the response was observed in 25 µM BAPTA treated cells (Fig. 5A). BMMC were treated with this suboptimal dose of BAPTA for 5 min prior to addition of 1×10^−6^ M PGE_2_. After 10 minutes, cytokines, PGE_2_ and BAPTA were removed and cells were cultured in cytokine-depleted medium for 20 h. BAPTA treatment alone did not alter the apoptosis induced by cytokine deprivation, but completely blocked the PGE_2_ mediated increase in apoptosis (Fig. 5B).

MAPK Erk 1/2 as well as p38 are activated by PGE_2_ [21]. PGE_2_ mediated activation of both MAPKs, assessed by phosphorylation at T202/Y204 and T180/Y182 respectively, is EP3 receptor dependent (Fig 5C and data not shown). Treatment of cells with PD98059, an inhibitor specific for kinase of Erk1/2, MEK1 kinase, blocked PGE_2_ induced T202/Y204 Erk 1/2 phosphorylation in a dose dependent manner (Fig 5D lower panel). Erk1/2 inhibition was sufficient to completely inhibit the increase in the number of 7-AAD stained cells in BMMC pre-treated with PGE_2_ prior to cytokine deprivation (Fig. 5D upper panel).

Activation of mast cells with 1×10^−6^ M PGE_2_ resulted in rapid Akt activation, measured by an increase in phosphoryplation at S473 (Fig. 5E upper panels). In EP3−/− BMMC, PGE_2_ induced phosphorylation of S473 was completely abolished (Fig. 5E lower panels). Treatment of WT mast cells with LY294002, an inhibitor of PI3 kinase responsible for activation of Akt, at concentration higher than 10 µM resulted in complete inhibition of PGE_2_ induced S473 phosphorylation. However, inhibition of Akt activation by LY294002 did not alter the ability of PGE_2_ to augment BMMC apoptosis (Fig. 5F).

### PGE_2_-stimulated MAPK activation and increase in apoptosis upon cytokine withdrawal is Calmodulin kinase II dependent

**Figure 6:**
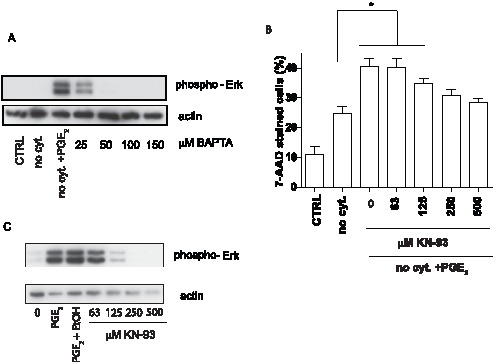
PGE_2_-stimulated MAPK activation is dependent on calcium and calmodulin dependent kinase II. **A**. Erk1/2 phosphorylation is inhibited by BAPTA. Cells were pretreated with various concentration of calcium inhibitor BAPTA before activation with 1×10^−6^ M PGE_2_, n = 3 independent experimetns of 1 culture of BMMC. **B**. Calmodulin dependent kinase II specific inhibitor KN-93 inhibited PGE_2_ increased apoptosis. BMMC were pretreated with various concentration of KN-93 for 10 min followed by activation with by 1×10^−6^ M PGE_2_. Dead cells stained with 7-AAD, n = 6 independent experiments of 3 cultures of BMMC. **C**. KN-93 at various concentrations inhibited Erk1/2 phosphorylation induced by 1×10^−6^ M PGE_2_, n = 4 independent experiments of 1 cultures of BMMC. BMMC cultured in complete medium (CTRL), medium without cytokines (no cyt.) and medium without cytokines in the presence 1×10^−6^ M PGE_2_ (no cyt. + PGE_2_). Statistical significance using ANOVA: * = P<0.05, ** = P<0.01, *** = P<0.001.

MAPK activation can be both calcium dependent and calcium independent. To determine the relationship between intracellular calcium mobilization and MAPK phosphorylation triggered by EP3 receptor, we treated BMMC with the intracellular calcium inhibitor BAPTA before addition of 1×10^−6^ M PGE_2_. Inhibition of intracellular calcium resulted in a complete inhibition of PGE_2_ induced Erk1/2 and p38 phosphorylation at BAPTA concentrations greater than 25 µM (Fig. 6A, data not shown). Erk 1/2 could be activated by intracellular calcium through several pathways including calcineurin and calmodulin dependent kinase II (CamKII) [22]–[24]. However, we found that activity of calcineurin in WT and EP3 −/− cells significantly decreased after PGE_2_ stimulation. Moreover a specific inhibitor of calcineurin, Cyclosporine A, was not able to inhibit Erk1/2 activation (data not shown).

To investigate a possible role of CamKII in the activation of MAPK in BMMC by PGE_2_, an inhibitor with specificity for CamKII, KN-93, was used. As shown in Fig. 6B, KN-93 effectively inhibited the PGE_2_ mediated increase in apoptosis during cytokine withdrawal in a concentration dependent manner ranging from 125 to 500 µM. At higher concentrations this agent was toxic to BMMC. The inhibition of CamKII with KN-93 correlated with inhibition of Erk 1/2 (Fig. 6C).

### PGE_2_ increases expression of Bim

**Figure 7:**
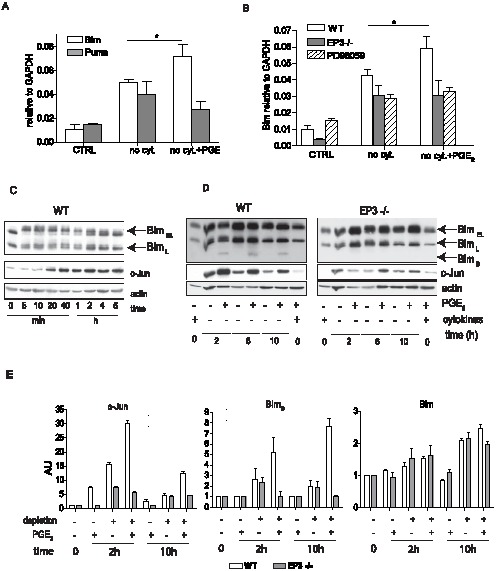
PGE_2_ increase Bim expression during cytokine deprivation. A. RNA was isolated from BMMC cultured in complete medium (CTRL), medium without cytokines (no cyt.) and medium without cytokines in the presence 1×10^−6^ M PGE_2_ (no cyt. + PGE_2_) for 10 h. Expression of Bim (*Bcl2l11*) and Puma (*Bbc3*), relative to GAPDH, was assessed by real time PCR using gene specific TaqMan Gene Expression Assay. B. RNA isolated as in A., Bim expression in WT, EP3−/−, and WT cells treated with ERK 1/2 inhibitor PD98059 in presence or absence of 1×10^−6^ M PGE_2_ during cytokine deprivation. C. Expression of Bim isoform and c-Jun in WT BMMC in complete medium after stimulation with 1×10^−6^ M PGE_2_ for indicated times. Representative of 3 independent experiments is shown. D. Expression of Bim isoforms and c-Jun various times after cytokine depletion in BMMC treated or untreated with 1×10^−6^ PGE_2_. 1 of 6 independent experiments is shown. E. Quantitative analysis of impact of cytokine withdrawal with or without PGE_2_ treatment on relative protein expression of c-Jun, Bim_S_ and total Bim in BMMC treated with 1×10^−6^ M PGE_2_ during cytokines withdrawal. Data are from panels shown in C and D sections of this figure as well as from additional experiments (not shown). AU-arbitrary units, n = 6 independent experiments of 2 culture of WT and EP3−/− BMMC. Student's two-tailed t test was used to evaluate statistical differences between cytokine deprive mast cells and cytokine deprived mast cells treated with PGE_2_ in A nad B. Statistical significance: * = P<0.05.

Our results suggested that PGE_2_ augmented apoptosis in mast cells through a signaling pathway that involves intracellular calcium release and Erk 1/2 phosphorylation. As shown in Fig.2 B, an increase in apoptosis is apparent at the mitochondrial level, suggesting that activated Erk 1/2, or events downstream of Erk 1/2 are able to upregulate intrinsic apoptotic pathway prior to MOMP. Bcl-2 family members Bim and Puma have been implicated in previous studies examining mast cells apoptosis induced by cytokine withdrawal [8]. Additionally, Erk1/2 was shown to regulate expression of c-Jun, an established positive regulator of Bim expression [25]–[27]. We therefore asked whether the activation of the Erk1/2 pathway by PGE_2_ was paralleled by alteration in the expression of genes known in these pathways. Expression of Bim and Puma in BMMC treated with either vehicle or PGE_2_ was assessed by qPCR 10 h after cytokine withdrawal. As expected, removal of cytokines from the culture medium led to an increase in expression of both Bim and Puma (genes *Bcl2l11*and *Bcc3*) compared to cells growing in complete medium (Fig. 7A). Pretreatment of BMMC with PGE_2_ for 20 min prior to cytokine withdrawal significantly increase Bim expression. In contrast, no difference between PGE_2_ and vehicle treated cultures was observed in the expression of Puma. Withdrawal of cytokines increased Bim expression also in EP3−/− cells; however, these cells were resistant to a further increase in Bim expression after PGE_2_ treatment (Fig. 7B). Inhibition of the Erk 1/2 pathway with PD98059 prior to PGE_2_ treatment, also prevent the increase in Bim expression (Fig. 7B). Previous studies have shown that PGE_2_ can induce apoptosis of lymphocytes, and that this response is dependent on c-Myc although it is Bcl-2 independent [28]. RNA from mast cells cultured in complete medium, mast cells depleted from cytokines for 10 h, and mast cells pretreated by 10^−6^ M PGE_2_ before 10 h depletion was analyzed for both c-Myc and Bcl-2 expression. Expression of both genes was significantly decreased in RNA samples prepared from cells after removal of cytokines. The presence of PGE_2_ did not prevent the decrease in either c-Myc or BCL-2 (data not showed). Therefore, PGE_2_ increase apoptosis induced by cytokine withdrawal is likely c-Myc and Bcl-2 independent.

To further delineate mechanism of Bim regulation, we evaluated changes in the protein levels of the various Bim isoforms and assessed cells for the appearance of c-Jun after PGE_2_ stimulation. c-Jun levels increased rapidly in mast cells in response to PGE_2_, with maximal level seen by 2 h (Fig. 7C). An increase was also seen after 2 h of cytokine withdrawal and the increase was greater in mast cells exposed to PGE_2_ (Fig. 7D, left). This augmented production of c-Jun was EP3 dependent as no observed increase in c-Jun was observed in lysates from EP3−/− cells after EP3 stimulation (Fig 7D, right panel). In the presence of cytokines only 2 isoform of Bim were identified in BMMC lysates (Bim _EL_ and Bim _L_) (Fig. 7C). 5 min stimulation with 1×10^−6^ M PGE_2_ lead to dramatic change in mobility of Bim _EL_ and Bim_L_ isoforms on western blot as both Bim isoforms showed multiple bands probably due to their phosphorylation (Fig. 7C). However, total Bim protein level did not significantly changed within 6 h of PGE_2_ activation (Fig. 7C). In the absence of cytokines, Bim protein level increased within 2 h of depletion and also low expression of Bim_S_ was apparent on long exposure of films (Fig. 7D,E). The presence of PGE_2_ resulted in a dramatic increase in the amount of Bim_S_ isoform 2–10 h after cytokine depletion in WT cells. 10 h after cytokine depletion, PGE_2_ stimulation also slightly increased levels of other Bim isoforms (Fig. 7E). Cytokine depletion of EP3−/−BMMC also resulted in increased levels of Bim, however, no further increase in expression of Bim was observed in cells exposed to PGE_2_ (Fig 7D,E). Together this suggest that cytokine withdrawal and EP3 activation work together to modulate the levels of Bim and to alter the ratios of the various Bim isoforms present in the mast cells.

## Discussion

**Figure 8:**
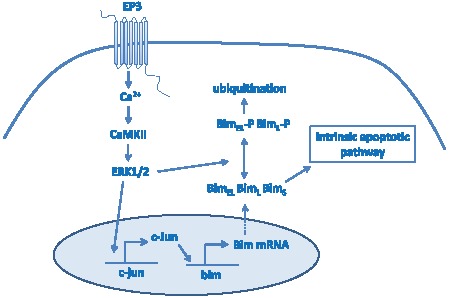
Schematic depicting of EP3 induced signaling pathway contribution to apoptosis during cytokine withdrawal. Withdrawal of cytokines results in activation of intrinsic apoptotic pathway due to imbalance between Bcl-2 anti-apoptotic protein family members and BH3 only pro-apoptotic proteins. Intrinsic apoptotic pathway in mast cells is upregulated by PGE_2_ via EP3 receptor and Ca^2+^-CaMII-ERK1/2 pathway. While Erk 1/2 mediated phosphorylation of Bim_EL_ and Bim _L_ immediately after PGE_2_ stimulation and this leads to ubiquination and degradation of these isoforms, Erk also induces expression of c-Jun, a potent upregulator of Bim expression. Final outcome of Erk regulation of Bim is a balance between Bim degradation after its phosphorylation and increase in its expression due to c-Jun. Data presented here support view that increase expression of Bim and proapoptotic action of EP3-Ca^2+^-CamKII –Erk1/2 pathway is prevalent in mast cells in presence of PGE_2_ and during cytokine withdrawal.

In this manuscript we analyzed the role of PGE_2_ in mast cell apoptosis during cytokine withdrawal. In our initial observation we found that exposure to PGE_2_ increases mast cell death during cytokine deprivation. However, PGE_2_ was not able to induce cell death in the presence of anti-apoptotic stimuli (grow factors and cytokines). This suggests that PGE_2_ is not an inducer of cell death, but rather it activates signaling pathway(s) which act in synergy with pro-apoptotic pathways triggered by withdrawal of cytokine. Analysis of the mitochondrial membrane potential, caspase-3 and late phases of apoptosis indicated that the higher number of dead cells in cultures treated with PGE_2_ during cytokine deprivation is caused by an increase in apoptosis. This further supports the synergy of PGE_2_-triggered and apoptotic pathways. Moreover, the up-regulation of all apoptotic events we analyzed suggested that the PGE_2_-triggered signaling pathway modulates apoptotic events before or during MOMP. Interestingly, pretreatment of BMMC with PGE_2_ shortly before cytokine deprivation, and subsequent absence of PGE_2_ during deprivation, was efficient in potentiation of apoptosis. This raises the possibility that pathways triggered by PGE_2_, or products of this pathway remained activated for a relatively long period of time. A possible explanation for these phenomenons is the induction of transcription of pro-apoptotic factors from Bcl-2 family by PGE_2_ which regulate MOMP. This is consistent with our overall finding that the mechanism by which the PGE_2_/EP3 pathway acts is increasing Bim transcription after cytokine withdrawal. This is also supported by the previous findings of others that cross-linking of FcεRI and activation of mast cells, before their depletion from cytokines induce Bim and modulate mast cell survival [29]. In neutrophils, GM-CSF treatment temporarily blocks apoptosis by inducing anti-apoptotic factors with rapid turnover and pro-apoptotic factors including Bim, which limit GM-CSF-mediated prolonged survival of neutrophils [30]. Binding sites for several transcription factors including Foxo, c-myb, and c-jun are present in the Bim promoter [31], [32]. While c-myb and c-jun can be activated by MAPK, Foxo expression is negatively regulated by Akt phosphorylation. It is interesting to speculate that upregulation of Foxo during cytokine withdrawal cooperates with induction of c-jun and/or c-myb to facilitate increased Bim transcription. Increased expression of c-jun and c-myb may be in part secondary to Erk activation by PGE_2_ (Fig.8). Bim undergoes alternative splicing to produce three splicing variants (Bim_EL_, Bim_L_ and Bim_S_). The smallest variant Bim_S_ is the most potent inducer of apoptosis [33]. Interestingly, Bim_S_ isoform was upregualated by EP3 dependent PGE_2_ activation as well as by cytokine withdrawal. PGE_2_ through EP3 receptor cause changes in mobility of Bim_L_ and Bim_EL_ proteins due to phosphorylation, which alter Bim binding capacity with anti-apoptotic members of Bcl-2 family as well as its degradation. Balance between degradation of phosphorylated Bim_EL_ and Bim_L_ isoforms and up regulated expression of all 3 isoforms by transcription factors is dramatically shifted during cytokine withdrawal of PGE_2_ treated BMMC toward increased expression and apoptosis.

Sustained intracellular calcium release has been reported as a pro-apoptotic stimulus in many cells [34]. An increase in intracellular calcium is also a distinguishing event during mast cell activation by PGE_2_ [16], [21]. Indeed, we found that calcium is required for both PGE_2_-triggered up-regulation of apoptosis and Erk1/2 activation in mast cells (Fig. 4 and 5). Interestingly, partial inhibition of intracellular calcium release (25 µM BAPTA) was sufficient to inhibit both upregulated apoptosis and Erk 1/2 phosphorylation (Fig. 4 and 5), suggesting a need for strong intracellular calcium activation. Although this data implies that intracellular calcium acts on apoptosis through activation of Erk 1/2, we cannot exclude the possibility that part of the increased apoptosis after PGE_2_ treatment is caused directly by intracellular calcium release. However, MEK inhibition of the PGE_2_-dependent increase in Bim expression makes this unlikely. Our data support a model in which EP3/calcium/CamKII/Erk/Bim signaling is the PGE_2_-triggered pathway responsible for increased apoptosis (Fig 8).

The role of Erk1/2 in cell survival is well characterized; however, recent evidence suggests that the activation of Erk1/2 also contributes to cell death. Erk 1/2 activation was shown to contribute to apoptosis by cisplatin [35], heavy ion irradiation therapy using a carbon beam [36], exposure to reactive oxygen species [37] or deprivation of pro-survival factors [38]. Erk1/2 can potentiate apoptosis by upregulation of caspase-3 activation, induction of p53 and other pro-apoptotic factor, expression of death ligands, and suppression of pro-survival activity of Akt (for review see [39]). Although these studies support the pro-apoptotic nature of Erk1/2 phosphorylation, the basis for Erk1/2 signaling in those events is not clear. It has been reported that prolonged Erk1/2 activation leads to a pro-apoptotic effect of Erk1/2 while transient activation protects against apoptosis. This is consistent with recent results obtained from mast cells deficient in SHP1 phosphatase, which is responsible for dephosphorylation of Erk1/2 [40]. Stimulation of cells with a low concentration of IL-3 protects against apoptosis in SHP1 negative cells, while a higher concentration of IL-3 promotes apoptosis and shows strong prolonged activation of Erk1/2. This suggests that the kinetics and duration of Erk1/2 activation might determine the effect of Erk1/2 on cell fate. Strong phosphorylation of Erk1/2, found after activation of mast cells with PGE_2_ supports this view.

Several previous reports have described the ability of PGE_2_ to either potentiate cell death [28], [41], [42] or protect against it [43], [44]. The contradictory outcomes reported likely reflect that the impact of PGE_2_ on cell death depends on cell types and death stimuli. Stimulation of EP2 and EP4 is mostly reported as anti-apoptotic, while stimulation of EP3 and EP1 are reported as pro-apoptotic or without an effect. It is likely that the relative expression of each EP receptor determines the final outcome of PGE_2_. For example, neutrophils express relatively more EP2 and EP4 receptors compared to EP3, while in BMMC, EP3 is the predominant EP receptor. Treatment of neutrophils with PGE_2_ protects them from apoptosis [45], [46], however treatment of BMMC and peritoneal mast cells by PGE_2_ as we report here up-regulates apoptosis. Similarly to mast cells, studies from other cells have found that the EP3 receptor augments apoptosis. In human neutrophils, a selective agonist of EP3, ONO-AE-248, induces death by disruption of mitochondrial potential without chromatin condensation, DNA fragmentation, or expression of PS on the plasma membrane [47]. EP3 was shown to mediate neurotoxicity on exposure to PGE_2_ during ischemia, or ischemic stroke through enhancement of inflammatory and apoptotic reactions in the ischemic cortex [41]. In colon carcinogenesis, EP3 plays an important role in suppression of cell growth and its down regulation enhances the late stages of this disease [48].

In summary, even brief and transient exposure to physiologically relevant levels of PGE_2_ can increase mast cell apoptosis during cytokine deprivation. We provide evidence that PGE_2_ mediates this action solely through the EP3 receptor. This increase in mast cell apoptosis is dependent on synergy between the EP3/calcium/CamKII/Erk pathway and intrinsic apoptotic pathways. Our analysis suggests that this pathway increases Bim expression and also may alter the relative levels of the various Bim isoforms, in particular the pro-apoptotic Bim_S_ isoform. The unique ability of PGE_2_ to increase apoptosis in mast cells could play an important role during resolution of allergic inflammation and suggest that while Nonsteroidal anti-inflammatory drugs (NSAID) can limit many symptoms of inflammation they also may prolong recovery in some instances.
